# Methane emissions and rumen metabolite concentrations in cattle fed two different silages

**DOI:** 10.1038/s41598-022-09108-w

**Published:** 2022-03-31

**Authors:** R. Bica, J. Palarea-Albaladejo, J. Lima, D. Uhrin, G. A. Miller, J. M. Bowen, D. Pacheco, A. Macrae, R. J. Dewhurst

**Affiliations:** 1 Scotland’s Rural College, SRUC, West Mains Rd, Edinburgh, EH9 3JG UK; 2 Royal (Dick) School of Veterinary Studies and the Roslin Institute, University of Edinburgh, Easter Bush Campus, Midlothian, EH25 9RG UK; 3 Biomathematics and Statistics Scotland, JCMB, Peter Guthrie Tait Road, The King’s Buildings, Edinburgh, EH9 3FD UK; 4 The University of Edinburgh, EaStCHEM School of Chemistry, The King’s Buildings, David Brewster Road, Edinburgh, EH9 3FJ UK; 5 AgResearch Grasslands Research Centre, Tennent Drive, 11 Dairy Farm Road, Palmerston North, 4442 New Zealand; 6 Present Address: Department of Computer Science, Applied Mathematics and Statistics, University of Girona, 17003 Girona, Spain; 7 Present Address: Institute National de La Recherche Agronomique (INRAE), 24 Chemin de Borde Rouge, 31320 Auzeville-Tolosane, France

**Keywords:** Animal physiology, Metabolomics

## Abstract

In this study, 18 animals were fed two forage-based diets: red clover (RC) and grass silage (GS), in a crossover-design experiment in which methane (CH_4_) emissions were recorded in respiration chambers. Rumen samples obtained through naso-gastric sampling tubes were analysed by NMR. Methane yield (g/kg DM) was significantly lower from animals fed RC (17.8 ± 3.17) compared to GS (21.2 ± 4.61) *p* = 0.008. In total 42 metabolites were identified, 6 showing significant differences between diets (acetate, propionate, butyrate, valerate, 3-phenylopropionate, and 2-hydroxyvalerate). Partial least squares discriminant analysis (PLS-DA) was used to assess which metabolites were more important to distinguish between diets and partial least squares (PLS) regressions were used to assess which metabolites were more strongly associated with the variation in CH_4_ emissions. Acetate, butyrate and propionate along with dimethylamine were important for the distinction between diets according to the PLS-DA results. PLS regression revealed that diet and dry matter intake are key factors to explain CH_4_ variation when included in the model. Additionally, PLS was conducted within diet, revealing that the association between metabolites and CH_4_ emissions can be conditioned by diet. These results provide new insights into the methylotrophic methanogenic pathway, confirming that metabolite profiles change according to diet composition, with consequences for CH_4_ emissions.

## Introduction

With growing demand for livestock products, cattle contribute significantly to enteric methane (CH_4_) emissions globally^1^. Cattle are estimated to produce between 250 and 500 L of CH_4_ per day^2^ with up to 90% of CH_4_ production occurring in the rumen^3^. The conversion of feedstuffs into volatile fatty acids (VFA), microbial protein and vitamins in the rumen involves a variety of different microbial species (including bacteria, archaea, protozoa and fungi, i.e. the rumen microbiota). During the enteric fermentation process, carbon dioxide (CO_2_) and hydrogen (H_2_) are also produced, and subsequently utilized by methanogenic archaea, producing CH_4_^4^.

Rumen methanogenic archaea mostly use the hydrogenotrophic pathway, with CO_2_ and H_2_ as precursors for CH_4_ production^5,6^. However, in addition to CO_2_ and H_2_, other compounds such as formate and methyl compounds (MA’s) can be precursors for CH_4_ production through the methylotrophic methanogenic pathway, particularly used by the novel archaea class *Thermoplasmata*^7^.

Rumen methylotrophs have lower H_2_ requirements, meaning they utilise 1 mol of H_2_ per mole of CH_4_ produced, compared to the 4 mol of H_2_ per mole of CH_4_ used by hydrogenotrophs^8^. However, methylotrophic organisms rely on availability of CH_3_-compounds, such as methanol and methylamines, as well as dissolved H_2_^9^.

Uronic acids are present at varying levels in different types of plants as constituents of polysaccharides, particularly pectin^10^; galacturonic acid is the main uronic acid in pectin^11^. Pectin is of interest in relation to CH_4_ formation because methanol is a major end product of pectin metabolism^12^. Studies have shown that pectin is generally present at lower concentrations in grasses when compared to legumes and fruit^13,14^. Methanol transformation, as well as degradation of betaine and choline, has been shown to be a precursor for methylamines (methylamine, dimethylamine and trimethylamine) which subsequently provide energy and carbon to the methylotrophic methanogens^7^.

*Thermoplasmata* utilise methylamines as their primary source of energy and carbon source in the production of CH_4_. Although it remains unclear whether *Thermoplasmata* can utilize the hydrogenotrophic methanogenic pathway as well, current data shows they belong to a separate niche compared to other rumen hydrogenotrophic methanogens^7^.

The amount and type of feed consumed by ruminants are the most important factors influencing CH_4_ emissions, but other factors such as variation between individuals, and geographical location also play a role in determining the overall emissions^2^. Approaches to mitigate CH_4_ emissions in ruminants usually focus on reducing H_2_ availability by modifying the diets given to the animal^2,7,15^.

The effects of legume based diets on ruminants have been tested in previous studies^16–18^, with results indicating that they lead to reduced CH_4_ emissions. Red clover (*Trifolium pratense,* RC) is one of the most common legumes grown in Western European countries, and can be used for grazing, pure or in mixtures, but is primarily used for silage production^19^. Red clover is richer in proteins and minerals, and has lower fibre and sugar contents than grass. Legume based diets are also associated with increased feed intake levels when compared to grass silages^20^. This characteristic of legumes is associated with higher rates of passage from the rumen when compared to grasses, thus decreasing the extent of rumen fermentation^21–23^.

Current methods to measure CH_4_ emissions such as SF_6_ and respiration chambers, the latter of the two considered the ‘gold standard’ in the field, tend to be costly, low throughput or invasive to the animal. This has prompted an increase in studies looking into proxies (indirect indicators/traits) to estimate CH_4_ emissions^24^. Rumen metabolites, such as VFAs, have the potential to be used as proxies for CH_4_ emissions, mostly because enteric fermentation of feed into VFAs is coupled with hydrogen release.

The development of advanced analytical techniques, such as nuclear magnetic resonance (NMR) spectroscopy and next generation sequencing allows for a more comprehensive description of the rumen biochemical network underlying CH_4_ production. NMR is a powerful analytical technique which can be used for the identification of metabolic biomarkers^25,26^.

The main objective of this study was to assess whether feeding red clover silage leads to decreased CH_4_ emissions, when compared to a grass silage diet, in beef cattle. We recognise that it would be necessary to test many examples of each silage type to confirm effects, so this work focusses on the relationship between rumen metabolites and methane production using G and RC silages to generate divergent metabolite profiles. Additionally, we explored which metabolites were found at different concentrations in the rumen of cattle offered the different diets, with particular focus on those associated with the methylotrophic pathway. The hypothesis was that steers fed the red clover silage (RC) would have lower CH_4_ emissions, due to increased DMI and subsequent increased rumen passage rate, and a greater number of metabolites associated with the methylotrophic pathway, due to the elevated pectin content, when compared to steers fed the grass silage (GS).

## Materials and methods

This study was conducted at the Beef Research Centre of SRUC (Edinburgh). The experiment was conducted in accordance with the requirements of the UK Animals (Scientific Procedures) Act 1986. The experiment was approved by the SRUC Animal Experiments Committee, which operates as the Local Ethical Review Group required under the UK Animals (Scientific Procedures) Act 1986. All methods are reported in accordance with the ARRIVE guidelines.

### Animals, experimental design and diets

The study was run for a period of 12 weeks (September – December 2017) and included 18 cross-bred steers that were 8–15 months old at the start of the study (mean initial weight = 348 ± 36.3 kg). Individual daily fresh weight intakes (kg/day) were recorded for each animal using electronic feeding equipment and dry matter intake (DMI; kg/day) subsequently calculated. Fresh water was provided ad libitum using a water trough, and diets were offered at approximately 1.05 times average daily intake to all steers using 14 electronic feeders (HOKO, Insentec, Marknesse, The Netherlands) to ensure ad libitum access to feed. All steers were bedded on wood fibre and sawdust to ensure that consumption of bedding did not contribute to nutrient intake and influence feeding behaviours. Steers were weighed weekly on a calibrated weigh scale. A changeover design was implemented with a 3-week adaptation period, with the cattle being subdivided into 3 groups of 6^27^. Within each group, animals were randomly allocated to either the red clover silage (RC) in the first run and grass silage (GS) in the second run (3/group) or vice versa, meaning 9 animals in total were fed RC and 9 GS. Each individual animal was allocated to the same respiration chamber when tested for both red clover and grass silage, to minimize effects due to differences in chambers and allow both the animal and the chamber to act as their own control.

Grass silage was from a primary growth cut on 01/06/2016, whilst RC silage was prepared from a regrowth (first cut date: 27/05/2017), with a cutting date of 20/08/2017. Red clover was ensiled in bales whereas GS was ensiled in a pit. The red clover was a 50:50 mixture of Merviot and Rozeta *Trifolium pratense* whereas the GS was composed of the germinal HSG 1 perennial ryegrass (*Lolium perenne*) mixture with the following varieties of ryegrass used in the mixture: AberZeus, AberWolf, AberGreen and AberGrain. Both swards were grown in fields adjacent to the Beef Research Centre (Edinburgh, UK). Wilting was undertaken for 28 h prior to ensiling, however wet weather conditions at that time meant that the silages were wetter than expected. The silage additive used for each silage at the commercially recommended rate was a mixture consisting of *Lactococcus lactis O-224* and *Lactobacillus buchneri* (SiloSolve FC, Chr-Hansen, Czech Republic). Minerals (Ca, Mg, P, Na) and vitamins (A, D3, E, B12), supplied by Downland Marketing Ltd., Carlisle, were added to the silages in powder form (100 g/head/day) prior to feeding, using a feed mixer wagon to ensure good distribution. Both diets were high in forage content and offered ad libitum to the animals (mixtures were: RC = 30 kg silage (fresh weight) + 100 g minerals; GS = 40 kg silage (fresh weight) + 100 g minerals). Both silages were of relatively low DM content, with poor fermentation characteristics, that is pH greater than 4.2, high levels of acetic acid and low levels of lactic acid (lactic 78 g/kg DM and acetic 30.55 g/kg DM for RC and lactic 76.3 g/kg DM and acetic 51.3 g/kg DM for GS)^28^. The chemical composition of the feeds is given in Table 1. Two bulked samples per diet type were taken: one from the first experimental period (23/10/17–15/11/17) and one from the second (27/11/17–20/12/17). Each bulked sample derived from grab samples obtained weekly throughout the study. Samples were analysed by wet chemistry for DM, CP, NDF, pH and ash by SRUC’s Analytical Service Department^29^. The determination of the lactic acid was done by high performance liquid chromatography (HPLC) and the VFA were determined via gas chromatography (GC)^28^; these analyse were conducted by Sciantec Analytical (Cawood, North Yorkshire, UK). One bulked sample per diet was taken for uronic acid analysis (100 g fresh weight for each sampling point, total 600 g fresh weight for RC and GS). Pectin was analysed as galacturonic acid, assuming 830 g galacturonic acid/kg pectin. Uronic acid analysis (Glucuronic, 4-O-Methyl-D-Glucuronic and Galacturonic) of the biomass hydrolysate was undertaken by the Celignis Limited (Limerick, Ireland) with ion chromatography equipment (Dionex ICS-3000), a PA-1 analytical column, and an eluent program involving a sodium acetate and sodium hydroxide gradient.

**Table 1:** Red clover and grass silage diet chemical composition and ingredients.

Diet	Red clover silage	Grass silage
DM (g/kg)	196	187
NDF (g/kg DM)	464	568
CP (g/kg DM)	158	138
Ash (g/kg DM)	110	74
pH	4.65	4.25
Acetic (g/kg DM)	30.5	51.3
Propionic (g/kg DM)	0.7	7
Butyric (g/kg DM)	3.1	4.4
Lactic (g/kg DM)	78	76
NH_3_-N (g/kg DM)	86	68
DM^1^ Uronics (g/kg)	199	207
GalU^2^ (g/kg DM)	63	21
Pectin^3^ (g/kg DM)	75	25

^1^DM content for samples selected for uronic acid analysis, ^2^Galacturonic acid, ^3^Pectin content determined following relationship denoted in Udén, (2018)^11^: Pectin = galacturonic acid/0.83.

### Respiration chamber operation and measurements

Methane emissions were measured using six open-circuit respiration chambers (No Pollution Industrial Systems Limited, UK)^30^. The total chamber volume is 73m^3^ and they were ventilated by recirculation fans set at 450 L/s. Temperature and humidity were set at 15 °C and 60% respectively and air was exhausted from the chambers at 50 L/s resulting in about 2.5 air changes/hour. Air flow was measured using in-line hot wire anemometers which were validated using a calibrated hand-held anemometer (Testo 417; Testo Limited, UK). Temperature, pressure and humidity were measured at 5-min intervals from the exhaust of each chamber and at the common air inlet ducts using calibrated loggers (PRHTemp101; Madgetech Inc., USA). The concentrations of CH_4_ were measured by IR absorption spectroscopy and the concentrations of H_2_ were measured using a chemical sensor (ADC MGA-3000 Multigas Analyser: Analytical Development Company Limited, UK). Zero and span calibrations were performed at the beginning and end of each group measurement period using gases of know concentrations. The gas concentrations in each chamber and inlet air were recorded every 6 min. Methane and H_2_ production were calculated using the difference between inlet and outlet gas concentrations multiplied by volumetric dry air flow and corrected to standard temperature and pressure (25 °C and 101,300 Pa).

To accustom the animals to the chamber environment they were moved to loose-housed single pens (4 × 3 m) with identical design to the pens within the respiration chambers. After 6 days of acclimatisation, steers were moved into the chambers and held there for a total period of 72 h, and CH_4_ and H_2_ were measured in the final 48 h of the chamber period. The cattle were fed once daily, and the individual animal daily feed intake was recorded.

Measurements from one steer could not be recorded as there were malfunctions in the chamber and hence no measurement was obtained, this animal was fed the RC diet. The final number of steers receiving the GS diet and the RC diet were 18 and 17 respectively. Therefore, the final dataset was comprised of 35 spectra.

### Rumen sampling and NMR data acquisition

Immediately after the animals (within a 2 h period) left the chambers, samples of ruminal fluid were obtained for each individual animal by inserting a naso-gastric tube (16 × 2700 mm Equivet Stomach Tube, Jørgen Kruuse A/S). This process took a total ~ 4 min for the 6 animals. To avoid saliva contamination, once the tube was removed it was cleaned thoroughly. The obtained fluid (approximately 50 ml) was filtered through 4 layers of muslin and stored at -20 °C until ready for analysis. Frozen samples were then thawed at room temperature 24 h before analysis. The samples for the metabolite analysis (2 ml) were centrifuged for 5 min at 13,793 rcf. The supernatant obtained underwent a further filtration using BD Plastipak 2 ml syringes and Whatman 0.2 µl filters. A final volume of 0.55 ml was obtained, and the final samples were used to acquire NMR spectra. The supernatant was transferred into 2 mL tubes, and phosphate buffer in D_2_O solution was added (50 μL) to a final concentration of 50 mM, and a final pH of 6.7 for all the processed samples. ^1^H-NMR spectra were acquired on a 600 MHz Avance III (Bruker, Karlsruhe, Germany) spectrometer equipped with a 5 mm TCI Z-gradient pulsed-field gradient (PFG) cryoprobe. Spectra were acquired at 27 °C using noesygppr1d Bruker pulse program and the following parameters: 64 transients and 4 steady state scans using a 4.0 s and 2.7 s relaxation and acquisition time respectively. Water suppression (γB_1_ = 50 Hz) was applied during the relaxation delay and a 10 ms NOESY mixing time. A spectral width of 20 ppm was used for collecting 64 k data points. Pulsed field gradients (1 ms) were applied at the end of the pre-saturation (50%) and the mixing time (-10%). The spectra were acquired in an automated mode within 7.5 min of active data acquisition. The shift in spectral signals was negligible across samples. More detail on the NMR analysis can be found in Bica et al*.*, (2020)^31^.

### Processing and profiling of spectra

The raw NMR spectra were analysed using Chenomx NMR Suite Version 8.5. Raw spectral Bruker FID files were imported directly into the Chenomx processor^32,33^. Processing involved automatic phase and baseline corrections, followed by manual adjustments to ensure that the spectra were all similar. Reference deconvolution (shim correction) was performed to fix any spectra imperfections and the DSS peak at 0.0 was used as reference and line broadening of 0.5 Hz was applied, in order to make the peaks more visible. Profiling was done using the Chenomx profiler module. Spectra were directly imported into the profiler module from the processor module. Both the sum line and subtraction line were used during the profiling to try to obtain the best fit for the identified metabolites, and targeted profiling was the technique implemented during the identification and quantification step^34^.

A total of 42 compounds were identified for all 35 spectra analysed. The metabolites identified were further confirmed by 2D ^1^H-^1^^3^C heteronuclear single quantum coherence spectroscopy (HSQC), which correlates the chemical shifts of protons and their directly attached carbon, and a 2D ^1^H-^1^H total correlation spectroscopy (TOCSY), with 20, 40 and 117 ms mixing times to monitor the correlation between all protons within individual spin system. A pooled sample deriving from 6 samples (3 RC and 3 GS) was used for this analysis. The sample was prepared according to Bingol et al*.*, (2016)^35^ and uploaded to the freely available Ohio State University COLMARm software (http://spin.ccic.osu.edu/index.php/colmarm/index2). COLMARm is a comprehensive metabolite identification software which used multiple two-dimensional NMR spectra to identify metabolites in complex mixtures^35^. The identification with COLMARm was cross-checked with the Human Metabolome Database (HMDB)^36^ and the raw 2D HSQC and TOCSY spectra obtained.

### Statistical analysis

The data analysis was conducted on the R studio system for statistical computing (v3.6.1) using the specialised packages *nlme*^37,38^ and *mixOmics*^39^. Statistical significance was determined at the usual 5% level (p < 0.05).

A comparison of the CH_4_ yield (CH_4_ g/kg DM) between the RC and GS diets was performed using a linear mixed model (LMM) fitted by restricted maximum likelihood (REML). The model included diet type as fixed effect, and chamber number and animal ID as random effects. This same LMM formulation was used to test for differences between diets for each of the metabolites individually, as well as the acetate:propionate (A:P) ratio, with the resulting p-values being adjusted for false discovery rate (FDR) to correct for the effect of multiple hypothesis testing^40^. Preliminary analysis revealed that spectra differed in intensity up to a fourfold and therefore a normalisation step was performed in order to make all the spectra comparable using the method based on a mixed model with the *hglm* package presented in Jauhiainen et al*.*, (2014)^41^.

Subsequently, metabolite profiles, resulting from the analysis of NMR data, were used as explanatory variables in a partial least squares discriminant analysis (PLS-DA) in order to investigate how they contributed to differentiate between the diets. The variable importance in projection method (VIP) was applied to assess the relevance of each metabolite considering the ordinary VIP > 1 threshold to identify the most important features^42–44^.

The ability of the metabolite profiles to explain the variability in the CH_4_ yield (g/kg DM) was assessed by four partial least squares (PLS) regressions models (with 2 latent components) including as explanatory variables the metabolites along with diet and DMI as covariates, only metabolites, and only metabolites within each diet type separately. A Venn diagram was used to summarise the overlap between the sets of metabolites considered important (VIP > 1) in the PLS analyses by diet type.

## Results

### Silage composition

The RC diet had higher content of CP and lower content of NDF when compared to GS; however the fermentation quality of both silages was determined to be low due to the fact that both butyric and acetic acid concentrations in the silages were higher and lactic acid concentrations were lower than is often observed (Table 1).

### Diet and methane emissions

**Figure 1:**
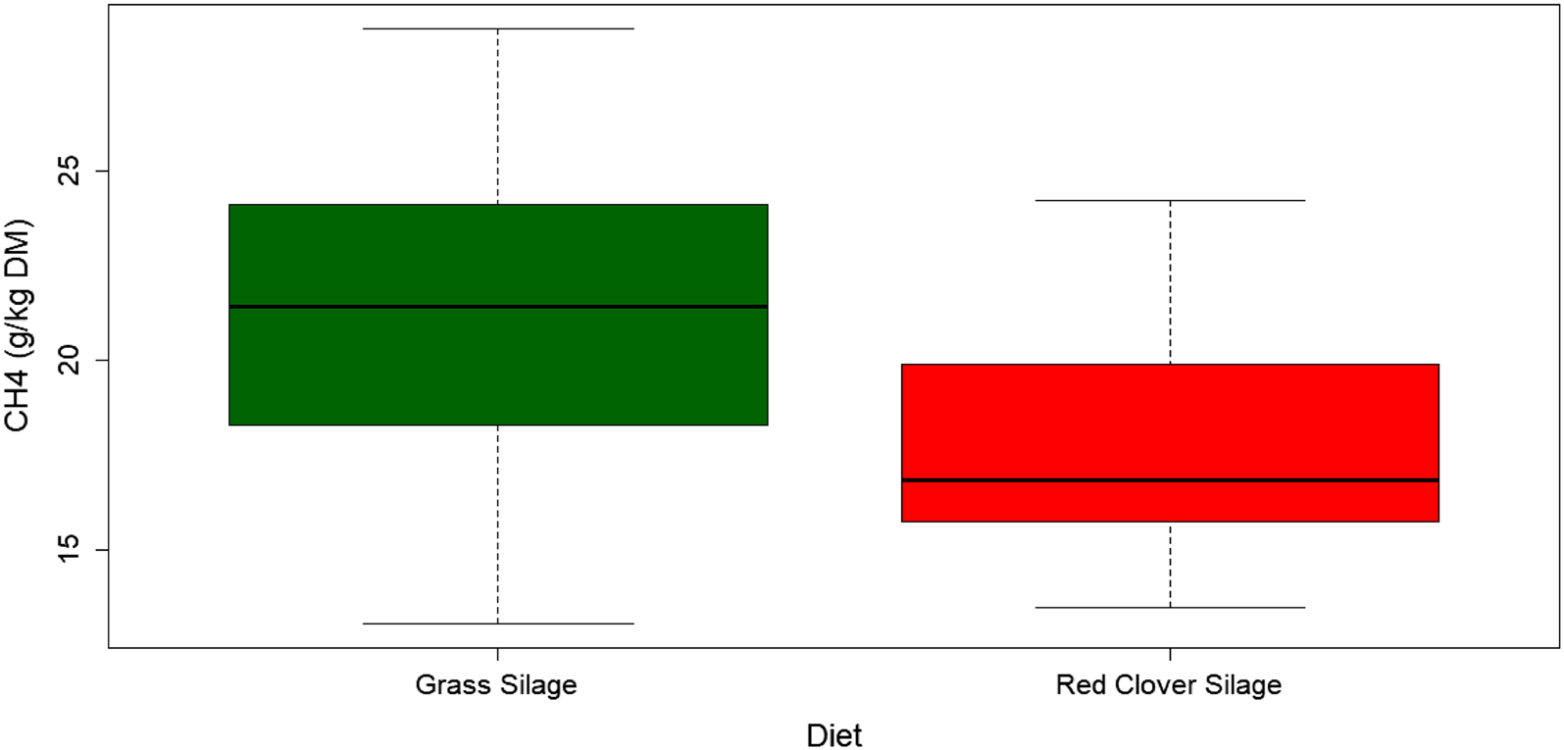
Boxplot of CH_4_ yield (CH_4_ g/kg DM) in grass silage and red clover silage fed animals.

Animals fed the GS diet had a significantly higher mean CH_4_ yield (g/kg DM intake; *p* = 0.008) than animals fed the RC diet (21.2 ± 4.61 and 17.8 ± 3.17 in g/kg DM units, respectively, Fig. 1).

Methane production (g/day) was numerically lower in the RC diet (122 g/d) than in the GS (133 g/d) (*p* = 0.1). Dry matter intake (DMI) for the RC and GS diets were 6.8 ± 0.97 kg/d and 6.5 ± 1.35 kg/d respectively.

### Comparison of rumen metabolite abundance by diet

Of the 42 metabolites identified in the study, 6 were significantly more abundant (p < 0.05) in animals fed the GS diet compared to those fed the RC diet according to the LMM results. These corresponded to the main VFA (acetate, propionate, butyrate and valerate), 3-phenylpropionate (3-PP) and 2-hydroxyvalerate.

Methylamine and methanol concentrations were higher in animals fed the GS diet, whereas dimethylamine and trimethylamine concentrations were higher in animals fed the RC diet, however, these differences were not significantly different. The complete list of identified compounds along with their mean values and standard deviations are shown in Table 2. Note that the (A:P) ratio was significantly different between the RC diet and GS diet (4.6 and 4.0 respectively, *p* = 0.02).

**Table 2:** Mean concentrations (µM) (± standard deviation) of rumen metabolites in samples taken from steers offered grass silage (GS) or red clover silage (RC) diets.

Metabolite	Red clover silage	Grass silage	^1^FDRpvalue
***VFA***			
Acetate*	52,120.8 ± 14,159.8	73,265.6 ± 16,616.7	0.000†
Butyrate*	4421.5 ± 1469.9	7718.7 ± 3857.4	0.028†
Isobutyrate*	803.5 ± 251.8	1119.4 ± 515.8	0.100
Isovalerate*	456.6 ± 135.7	612.3 ± 243.8	0.100
Propionate*	11,449.1 ± 3832.0	18,722.8 ± 5408.5	0.001†
Valerate*	615.2 ± 235.6	1298.7 ± 694.1	0.009†
***Methyl compounds***			
Dimethylamine	2.02 ± 1.21	1.86 ± 1.7	0.795
Methylamine*	12.5 ± 13.3	15.6 ± 15.7	0.795
Methanol*	23.1 ± 5.5	25.3 ± 7.5	0.483
Trimethylamine*	1.91 ± 0.4	1.69 ± 0.5	0.136
***AA***			
3-Phenylpropionate*	169.3 ± 102.9	416.0 ± 120.4	0.000†
Alanine*	60.9 ± 140.2	64.4 ± 95.4	0.805
Alloisoleucine	22.8 ± 17.8	20.9 ± 10.7	0.800
Aspartate	32.8 ± 20.8	34.1 ± 26.3	0.805
Benzoate*	22.1 ± 13.7	33.5 ± 13.4	0.061
Betaine	7.38 ± 13.2	6.71 ± 9.5	0.800
Creatine	6.51 ± 2.8	8.98 ± 7.5	0.278
Creatinine	6.49 ± 2.1	6.7 ± 2.4	0.805
Glutamate*	67.5 ± 35.7	72.5 ± 4	0.800
Glycine*	47.4 ± 62.1	194.2 ± 604.7	0.656
Isoleucine*	21.6 ± 15.0	21.7 ± 17.6	0.968
Leucine*	38.1 ± 20.7	45.1 ± 25.6	0.278
Phenylacetate*	185.9 ± 70.4	177.8 ± 105.2	0.849
Valine*	27.7 ± 35.6	24.9 ± 29.5	0.696
***Nucleotide metabolism***			
Uracil*	8.75 ± 5.1	11.1 ± 5.1	0.120
Xanthine	7.78 ± 1.97	9.21 ± 2.2	0.101
***Carbohydrates***			
Glucose*	74.3 ± 60.3	76.4 ± 54.8	0.932
Maltose*	14.9 ± 11.2	25.3 ± 27.3	0.344
Fructose	17.7 ± 12.5	27.1 ± 23.6	0.120
***Miscellaneous***			
1,3-Dihydroxyacetone	9.22 ± 5.6	8.3 ± 6.3	0.800
2-Hydroxyvalerate	87.7 ± 32.6	223. ± 129.6	0.006†
3-Hydroxyphenylacetate	15.0 ± 8.0	19.7 ± 5.1	0.076
Acetamide	12.6 ± 5.3	18.7 ± 9.6	0.056
Acetoacetate	10.7 ± 7.5	13.3 ± 16.3	0.696
Caffeine	1.88 ± 1.8	2.1 ± 2.5	0.805
Choline	4.17 ± 1.8	5.2 ± 5.8	0.696
Ethanol*	123.4 ± 70.0	141.5 ± 83.5	0.656
Formate	96.2 ± 19.3	115.2 ± 32.3	0.138
Fumarate	1.89 ± 1.3	2.13 ± 1.1	0.795
Lactate*	99.1 ± 186.0	132.7 ± 315.9	0.656
Malonate	6.61 ± 8.1	6.23 ± 3.6	0.872
Succinate*	34.1 ± 18.8	36.2 ± 36.1	0.842

^1^fdrpvalue = p-value adjusted by false discovery rate (Benjamini and Hochberg, 1995). *metabolite identities confirmed by 2D HSQC-TOCSY spectra analysis (see supplementary Table 1Sand Fig. 1S). †statistically significant differences noted between diets.

### Differentiation between diets by metabolite profiles

**Figure 2:**
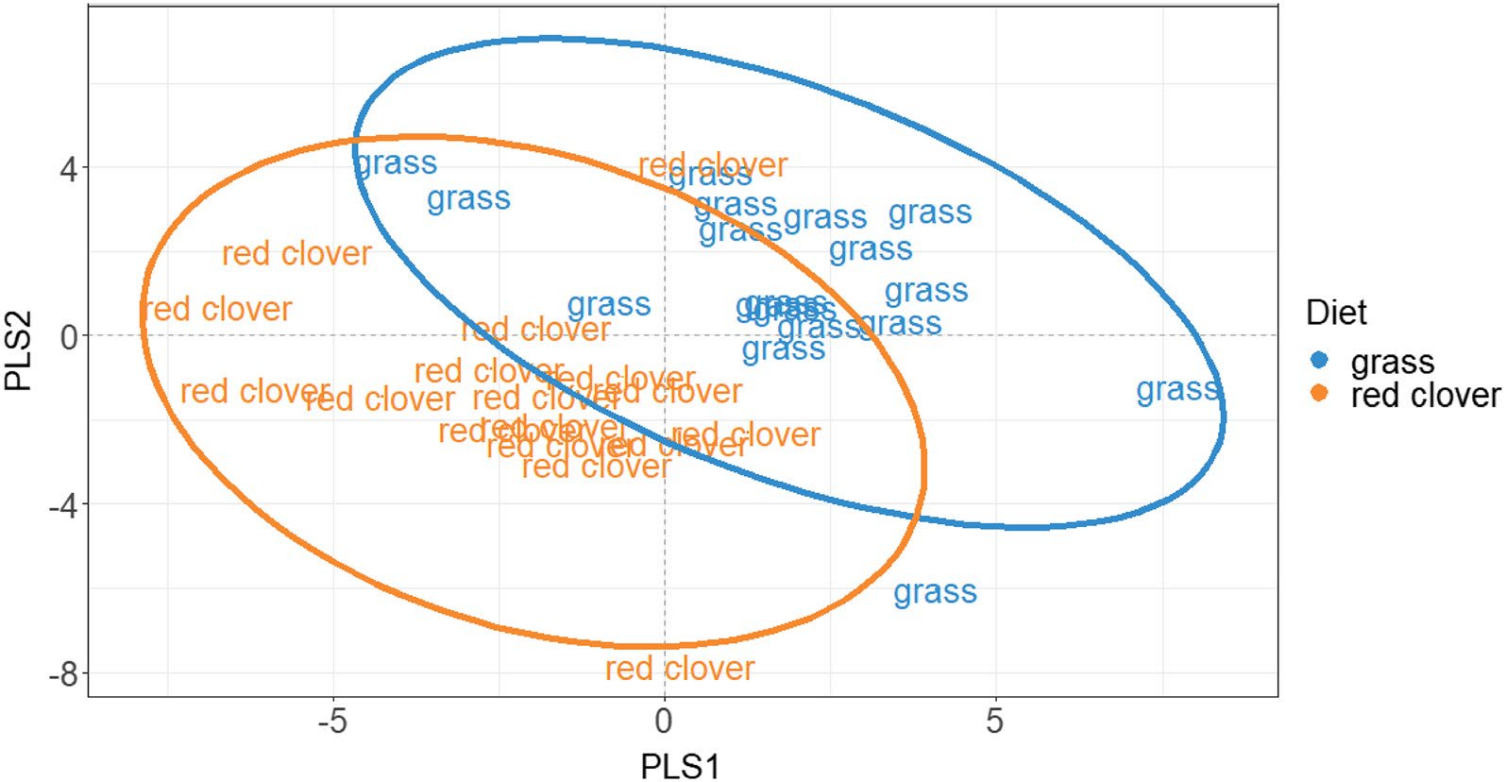
Partial least squares discriminant analysis (PLS-DA) score plots of 35 rumen samples from the animals fed grass silage and red clover silage.

Figure 2 displays the PLS-DA score plot based on the first two PLS components (PLS1 and PLS2; 51% of between-group variation explained). It shows a marked distinction between metabolic profiles of the animals fed GS (in blue) and the animals fed RC (in orange) along the direction of the second diagonal (Fig. 2).

According to the VIP criterion^44^, the most important metabolites for the distinction between animals fed the different diets (VIP > 1) included the 6 metabolites that showed significant differences in the LMM modelling (2-hydroxyvalerate, propionate, valerate, butyrate, acetate, 3-PP), as well as benzoate, isoleucine, dimethylamine, aspartate, 3-hydroxphenylacetate, iso-valerate, iso-butyrate, phenylacetate, valine and glutamate (Table 3).

**Table 3:** Partial least squares discriminant analysis (PLS-DA) variable importance of projection (VIP) values showing which of the 42 metabolites were more important for the distinction between animals fed RC and GS.

Metabolites	VIP
2-Hydroxyvalerate	1.77
Propionate	1.59
Valerate	1.56
Butyrate	1.46
Acetate	1.46
Benzoate	1.32
3-Phenylpropionate	1.30
Isoleucine	1.28
Dimethylamine	1.15
Aspartate	1.15
3-Hydroxyphenylacetate	1.09
Isovalerate	1.09
Isobutyrate	1.09
Phenylacetate	1.02
Valine	1.02
Glutamate	1.01
Acetamide	0.97
Leucine	0.96
Xanthine	0.95
Succinate	0.93
Malonate	0.91
Trimethylamine	0.91
Glucose	0.89
Acetoacetate	0.88
1,3-Dihydroxyacetone	0.88
Formate	0.86
Ethanol	0.86
Fructose	0.82
Alanine	0.78
Alloisoleucine	0.77
Fumarate	0.75
Maltose	0.73
Creatine	0.72
Uracil	0.72
Choline	0.68
Methanol	0.67
Lactate	0.60
Caffeine	0.48
Glycine	0.46
Methylamine	0.40
Betaine	0.31
Creatinine	0.16

Most of the 16 metabolites with a VIP > 1 were more abundant in samples from steers offered the GS diet (acetate, 2-hydroxyvalerate, 3-phenylpropionate, butyrate, valerate, propionate). However, valine and phenylacetate were more abundant in samples from steers offered the RC diet.

### Modelling methane from rumen metabolites and covariates

Four PLS regression models were fitted to estimate CH_4_ from different sets of explanatory variables: 1) (Model 1) metabolites, diet and DMI as covariates, 2); (Model 2) only metabolites, 3); (Model 3) only metabolites using the RC diet data, 4); and (Model 4) only metabolites using the GS diet data. Model 1 explained a higher percentage of the CH_4_ variation than Model 2 (R^2^ = 0.65 and R^2^ = 0.52, respectively). Overall, the lists of most important metabolites in Models 1 and 2 (VIP > 1) were similar (indicated in light blue in Table 4); with diet and DMI also being major contributors in Model 1. Of the three main VFA (acetate, butyrate, propionate) only butyrate was within the metabolites that had a VIP > 1 for both Models 1 and 2. Propionate and acetate did not appear to contribute significantly for the prediction of CH_4_ (VIP of 0.75 and 0.52 respectively for Model 1, and 0.84 and 0.57 respectively for Model 2). Of the methyl compounds, only trimethylamine had a VIP > 1.

**Table 4:** Partial least squares (PLS) variable importance in projection (VIP) values for the individual models. Light blue indicated VIP > 1.

Model	1^1^	2^2^	3^3^	4^4^
***External cofactors***				
DMI	2.30	N/A	N/A	N/A
Diet	1.43	N/A	N/A	N/A
***VFA***				
Acetate	0.52	0.57	0.71	0.44
Butyrate	1.34	1.48	0.97	1.33
Isobutyrate	0.86	0.96	0.56	1.17
Isovalerate	0.76	0.86	0.68	1.15
Propionate	0.75	0.84	1.10	0.60
Valerate	0.76	0.85	0.74	0.71
***Methyl compounds***				
Methanol	0.86	0.89	1.45	1.02
Dimethylamine	0.95	0.99	0.98	0.66
Methylamine	0.29	0.34	0.96	0.52
Trimethylamine	2.02	2.20	0.56	2.05
***AA***				
3-Phenylpropionate	1.04	1.18	2.23	0.74
Alanine	0.83	0.88	0.82	0.97
Alloisoleucine	0.77	0.82	0.61	0.87
Aspartate	1.07	1.09	1.09	1.00
Benzoate	0.25	0.25	0.41	0.58
Betaine	0.93	0.98	1.05	0.76
Creatine	0.81	0.89	0.98	0.93
Creatinine	0.82	0.93	0.70	0.77
Glutamate	1.07	1.10	1.08	0.97
Glycine	0.76	0.88	0.94	1.11
Isoleucine	0.72	0.71	0.77	0.95
Leucine	0.81	0.83	1.02	0.92
Phenylacetate	0.62	0.68	0.81	1.28
Valine	1.29	1.33	0.96	1.11
***Nucleotide metabolism***				
Uracil	0.56	0.62	0.75	0.51
Xanthine	0.42	0.45	0.51	0.82
***Carbohydrates***				
Glucose	1.36	1.43	1.49	1.01
Fructose	0.67	0.71	0.59	0.94
Maltose	0.53	0.51	0.15	1.02
***Miscellaneous***				
1,3-Dihydroxyacetone	1.03	1.07	1.14	0.51
2-Hydroxyvalerate	0.90	1.00	0.42	0.82
3-Hydroxyphenylacetate	0.20	0.23	0.46	1.12
Acetamide	1.56	1.74	1.90	1.35
Acetoacetate	0.78	0.79	0.92	1.07
Caffeine	0.65	0.68	0.77	0.62
Choline	1.25	1.29	0.69	1.19
Ethanol	0.85	0.88	1.37	1.03
Formate	0.68	0.79	1.12	1.11
Fumarate	1.04	1.08	0.69	1.32
Lactate	0.78	0.83	1.13	0.89
Malonate	1.14	1.18	0.88	0.97
Succinate	1.21	1.24	1.49	1.23

^1^Model using metabolites + DMI + Diet, ^2^Model using only metabolites, ^3^Model using only metabolites with RC diet data, ^4^Model using only metabolites with GS diet data.

**Figure 3:**
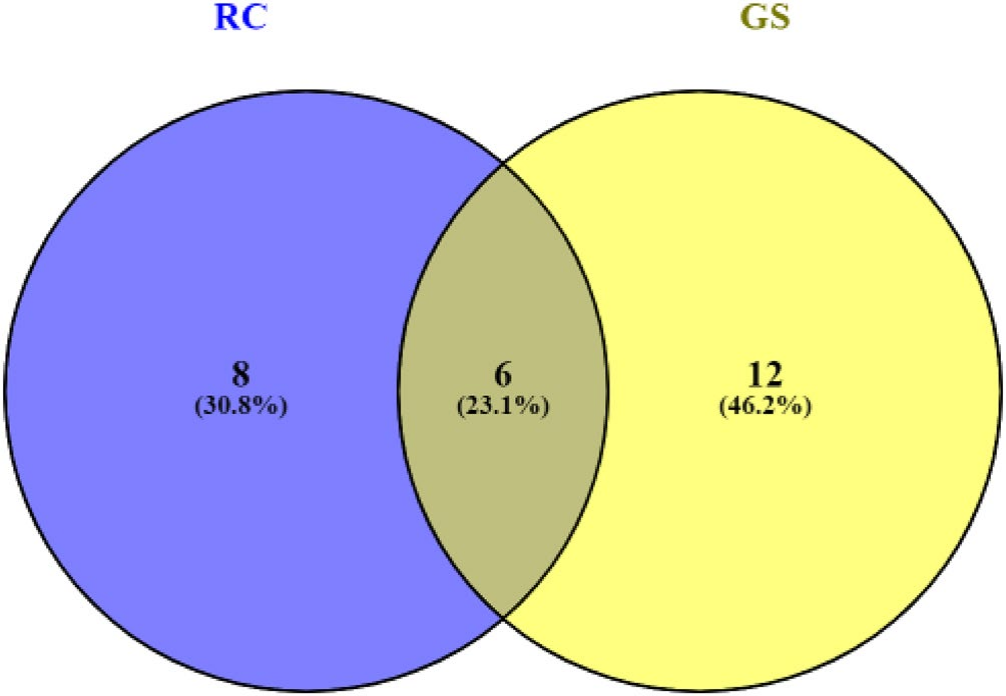
Venn diagram of number of metabolites which were relevant according to the VIP criterion in PLS modelling between Model 3 and Model 4 for red clover silage (RC) and grass silage (GS). The common metabolites were acetamide, succinate, glucose, methanol, ethanol and formate.

As to the PLS models by diet type when comparing Models 3 and 4, (only metabolites within diet), the R^2^ were 0.77 and 0.68 for RC and GS, respectively. Sets of 14 and 18 metabolites respectively for Model 3 and 4, were identified as important (VIP > 1). Of this total, 6 were similar between both models (Fig. 3): acetamide, succinate, glucose, methanol, ethanol and formate.

Regarding the PLS models calculated within diets (Model 3 & 4), the VIP values show that propionate was an important predictor within RC-fed animals, whereas butyrate was important within GS-fed animals (although note that it was only marginally below the VIP threshold in the case of RC). With regards to the most important variables in each model, 3-PP had the higher VIP for RC-fed animals, whereas it was trimethylamine for the GS-fed animals. Regarding the remaining methyl compounds, methanol had a VIP > 1 in both models, whereas both methylamine and dimethylamine had VIP < 1 in both models (although only marginally so in the case of RC).

Finally, note that a reduced PLS regression model of CH_4_ emissions with only methyl compounds (methanol, methylamine, di- and trimethylamine) as explanatory variables was fitted (data not shown). The results showed that only trimethylamine was considered important according to the VIP criterion (VIP = 1.59), whereas dimethylamine, methanol and methylamine were not (VIPs of 0.83, 0.51 and 0.48 respectively), showing a relative ordination comparable to the full models described above.

## Discussion

The present study used NMR to assess the rumen metabolite profiles of beef cattle offered diets consisting of two different silages. The main objectives of this study were to assess how a specific red clover silage diet affects CH_4_ emissions and to clarify the influence of these two silages on the rumen metabolite composition, with focus on whether our selected RC silage diet leads to increased concentrations of the metabolites associated with the methylotrophic methanogenic pathway, namely methanol, methylamine, dimethylamine and trimethylamine.

Both silages were first cut materials prepared in 2016 and stored for an extended period until 2017. The red clover silage had a higher CP content and lower NDF content when compared to the GS, in agreement with many other studies in the literature^45,46^. The relatively high acetic acid concentrations in these silages may be related to the long storage period and high moisture content of the silages (< 20% DM) particularly for the GS used in the study. Previous studies showed that wet silages which are ensiled for extended periods of time may lead to elevated levels of acetic acid and NH_3_-N^47,48^. Butyric acid concentrations were high in both silages (> 0.5% DM) caused by the high moisture content, possibly indicating clostridial fermentation which may explain the low quality of the silages^48^. The reason for the low quality silages is attributed to a combination of factors including the wet year of harvest and some mould generation occurring which led to the poor fermentation of the silages and subsequently reduced their quality.

With CH_4_ production by ruminants being a large contributor to the global greenhouse gas budget, and with ruminant production systems being the largest contributors to CH_4_ emissions by the livestock sector (total enteric CH_4_ emissions of the sector are 2.7 gigatonnes CO_2_ -eq per year) understanding the underlying metabolic mechanism/biochemical network is important^49–51^. The three main approaches taken to reduce CH_4_ emissions are 1) to change the diet composition which will alter VFA production, reducing the available H_2_ produced during enteric fermentation; 2) increasing the feed passage rate through the rumen, altering the extent of rumen fermentation and VFA production patterns; and 3) feeding high quality diets, thus decreasing CH_4_ emissions in relation to productivity^52^. In the current study, CH_4_ yield (g/kg DMI) was significantly lower when animals were offered the RC silage compared to the GS. Previous studies have shown decreased CH_4_ emissions from animals fed diets based on legumes in comparison to grasses^50^, though this has not always been noted^53^. The inconsistencies between results in the literature may be due to differences in forage composition (effects of stage of maturity or the presence of tannins in some legumes), silage quality or to between-animal variation^52^. In the study by van Dorland et al*.*, (2007)^53^, which compared red and white clovers mixed with ryegrass, no significant difference was observed in CH_4_ emissions and this was attributed to the low clover cotent within the diet (25–30%). However, in the current study, red clover made up a much higher proportion of the diet.

Waghorn et al*.,* (2002)^17^ reported higher feed intakes for young ram lambs offered a legume-based diet in comparison to grass-based forages (1.76 kg/day and 1.16 kg/day, respectively). Similarly, Lüscher et al*.*, (2014)^54^ showed increased DM intake in sheep offered legumes as either silages, hay or fresh herbages when compared to grasses. Additionally, Dewhurst et al*.,* (2003)^55^ noted an increase in DM intake of between 2–3 kg/day with red and white clover silages when compared to perennial ryegrass silages offered to dairy cattle. However, in the current work no significant difference was noted bewteen RC and GS, possibly attributed to the quality of the silages which hindered the intake potential of the RC diet.

Legumes result in higher rates of passage from the rumen, potentially leading to a shift of the site of digestion from the rumen to the intestines which would ultimately reduce the extent of fermentation in the rumen, leading to lower CH_4_ emissions^53,55^. Furthermore, Waghorn et al*.,* (1989) demonstrated higher rates of passage from the rumen of Friesian × Jersey cows when fed lucerne (*Medicago sativa L.*) when compared to fresh ryegrass (*Lolium perenne L.*). It seems likely that a reduction in the proportion of digestion occurring in the rumen explains some of the reduction in CH_4_ yield from RC when compared to the GS diet^56^.

It is well established that modifying diets fed to ruminants leads to a change in CH_4_ emissions and alterations in the VFA proportions^47^. Acetic, propionic and butyric acid are the predominant VFA produced in the rumen, and their concentrations may vary depending on feed intake, pH, diet composition and passage rates^57^. Diets that shift the rumen to a propionate dominated fermentation have been associated with reductions in CH_4_ emissions, whereas diets that shift to a predominantly acetate/butyrate dominated fermentation are associated with increased CH_4_ production^58^. The fermentation of feedstuffs into acetic and/or butyric acid generates excess hydrogen, which is then utilised by methanogens for CH_4_ production, whereas fermentation into propionic acid utilises hydrogen – so that less hydrogen is available for hydrogenotrophic methanogenesis^15^. In the current study, the main VFA acetate, butyrate and propionate had significantly lower concentrations in samples from steers offered the RC diet, confirming the suggestion above that higher rumen passage rates reduce the extent of rumen fermentation. Similarly, the significantly higher A:P ratio observed in the RC diet suggests that a reduction in the extent, rather than the type (acetate or propionate driven), of rumen fermentation explains the reduction in CH_4_ yield from animals offered RC.

Of the other metabolites identified, 3-phenylpropionate (3-PP) was significantly more abundant in rumen fluid from steers offered the GS diet compared to RC (416 µM and 169 µM, respectively, p < 0.01). The presence of 3-PP has been previously reported as essential for adherence and degradation of cellulose by *R. albus*, potentially improving fermentation of the GS diet^59^. It has also been suggested that 3-PP and phenylacetate have an inverse relationship, as one increases in concentration, the other one drops. This was observed in the current study, with 3-PP being present in high concentrations in rumen samples from animals offered the GS diet, whereas phenylacetate was present in greater concentrations (numerically) for the RC diet. Both these compounds have been previously identified in the aromatic region of a NMR spectra^43^. In a study by O’Callaghan et al*.*, (2018)^60^ looking at pasture fed dairy cattle with ryegrass or a ryegrass-white clover mixture, it was noted that 3-PP was more prevalent in samples from cattle offered the ryegrass diet, whereas phenylacetate was more prevalent in those offered the ryegrass-white clover mix diet. Both 3-PP and phenylacetate derive from the activity of ruminal microbiota on plant constituents, through the hydrogenation of phenolic compounds^43,60^. Martin et al*.,* (1983)^61^ also proposed an altenative origin of these compounds is through the deamination of aromatic amino acids such as tyrosine and phenylalanine. However, more research is necessary to further elucidate the relationship between these two metabolites.

Regarding the remaining amino acids identified in the current work (Table 2), no significant differences were noted in their concentrations between diets. Previous studies have shown that the higher levels of crude protein (CP) associated with legume based diets tends to lead to an increase in amino acid content in the rumen, as they provide proteinaceous substrate for microbial degradation^60,62^. However, this was not found in the current study, and this was suggested to be attributed to the high passage rate of the feed in the rumen of the RC diet. Dewhurst et al*.*, (2003)^55^ showed how when comparing grass and legume silages, namely white clover, red clover, and lucerne (*Medicago sativa*), red clover silage behaved similarly to grass silage, in terms of rumen passage rates, when compared to the other two silages. This may also help explain why significant differences were not noted at the rumen amino acid level.

In the current study, pectin was measured as galacturonic acid content, taking into account the relationship described by Udén (2018) & Bucher, (1984)^11,63^. Pectin was more abundant in the RC diet compared to the GS diet (75 g/kg DM and 25 g/kg DM), in agreement with previous studies. Beever et al*.*, (1985)^64^ reported a higher pectin content in the white clover compared to ryegrass (4.45 and 0.83 g/kg DM, respectively). Waghorn (1986)^65^ compared the content of pectin in red clover, fresh lucerne and lucerne hay, and reported similar content of pectin in red clover and lucerne hay (67 g/kg DM), and a greater content in fresh lucerne (69 g/kg DM). Additionally, Waghorn et al*.*, (2006)^13^ concluded that pectin content in legumes may account for approximately 10–12% DM content, and that it can be used as a precursor for methylamines and ultimately lead to CH_4_ production.

Pectin digestion in the rumen involves the removal of the methoxyl group through hydrolysis of methyl esters, with subsequent formation of methanol^66^. Betaine and choline are found in many plants, and therefore present in ruminant diets^43,67^; betaine is important in the methyl group metabolism^67^, and choline is a component of plant membrane material ingested by animals^68^. Methanol, betaine and choline are converted into methyl compounds (methylamine, dimethylamine and trimethylamine) in the rumen^9,42^. Poulsen et al*.*, (2013)^7^ suggested a similar mechanism, indicating that novel methylotrophic methanogens of the *Thermoplasmata* genus utilise choline, betaine, and methanol as degradation substrates which are a carbon source for di- and trimethylamine. Zhao et al*.*, (2014)^69^ also suggested that the degradation of choline, phenylalanine and other dietary components may be important for the production of methylamine^70^. Mitchell et al., (1979) and Neill et al*.,* (1978), also showed that both betaine and choline methyl groups are rapidly metabolized into methyl compounds, and subsequently into CH_4_ through the methylotrophic methanogenic pathway^65,66^.

In the present study, methanol, choline and methylamine were more abundant in the rumen fluid from animals offered the GS diet than in their RC-fed counterparts, whereas trimethylamine, dimethylamine and betaine were more abundant when animals were offered the RC diet, however, these differences were not significant. Despite these differences being solely numerical, it was speculated that the decreased concentration of methanol and choline and simultaneously increased concentration of di- and trimethylamine for the RC diet may be explained by increased degradation of these products to benefit the formation of di- and trimethylamine. Another suggested mechanism is that methanol may be lost through the ensiling process in the form of ‘green odour’^71^. This may be caused by degree of pectin methylation (addition of a methyl group to a substrate) which may be responsible for increased methanol production, and subsequently increased methanol losses^11,72,73^. However, the direct link between pectin methylation and methanol release in silages is yet to be quantified.

Animals offered diets differing in legume content will supply the rumen microbiome with different substrates for fermentation, leading to varying metabolic profiles, as confirmed in this study. Diet composition and DMI were found to be the most important factors explaining variation in CH_4_ emissions in the PLS analysis (VIPs of 1.4 and 2.3, respectively), in agreement with previous studies^74,75^. Accordingly, the model including diet composition and DMI along with the metabolites as explanatory variables reached a higher percentage of explained variation of CH_4_ yield than when including exclusively metabolites.

Although most VFAs were found to be significantly different between RC and GS diets in the study, our results confer butyrate a leading role. According to the literature, the main VFA related to CH_4_ is acetate^15,31^ and it has been shown by Williams et al*.,* (2019)^75^ that VFA’s alone should be able to predict CH_4_ emissions quite well without additional external factors. For the RC diet, propionate was found to be the most important VFA explaining variation in CH_4_, whereas it was butyrate for the GS diet. This agrees with past studies, as of the main VFA, propionate is generally associated with diets that are lower in CH_4_, whereas butyrate and acetate are associated with high CH_4_ yield diets^15,76^. Methane emissions are also generally related to the ratio of acetate to propionate (A:P), with decreasing CH_4_ production associated with decreasing A:P ratio^15^. The relevance of the interplay between relative abundance of these three VFA in relation to CH_4_ yield has been determined in previous work^6^. In the current study, as previously discussed, the A:P ratio was higher in the RC diet. Therefore, as well as suggesting that in the current case it was not the type of fermentation that was important, but the extent of fermentation we also attribute these inconsistencies due to the low quality of the silage feeds used in this study. Thus, highlighting the impact that silage quality may have on fermentation characteristics as well as CH_4_ emissions in ruminants.

As to the 6 common metabolites identified as important in the separate analyses by diet type (Fig. 3), all were present in higher concentrations in samples from animals offered the GS diet. Of these, formate and succinate have been described as important fermentation products of cultured rumen bacteria^60,77^. Formate is metabolized rapidly in the rumen and can be converted into H_2_, which when coupled with CO_2_ can lead to increased CH_4_ production in the rumen^76^. It has been reported that rumen methanogens may be able to directly metabolize formate to produce CH_4_ in the rumen^78,79^. However, no differences in formate were observed and therefore no further conclusions can be drawn. Succinate is produced as a by-product of rumen fermentation and is decarboxylated by rumen enzymes to form propionate^60^. These two together may explain the fermentation characteristics observed in the current study. Further work is, however, required to confirm this.

Regarding the methyl compounds, trimethylamine was considered important to explain CH_4_ variation within the GS diet and when considering both diets simultaneously. The observation that trimethylamine was less important for the prediction of CH_4_ in animals offered the RC diet indicates that the influence of the metabolites on the CH_4_ emissions differs according to the diet. Additionally, methanol was identified as one of the most important metabolites from the separate models by diet. Other rumen metabolites that were identified as important in the present study, notably glucose, aspartate, 3-PP and formate, have been associated with CH_4_ production in previous studies^28,43^.

In conclusion, this study showed how both CH_4_ and rumen metabolites can differ between two low-quality silages. We also showed how the changes in CH_4_ emissions observed may be more related to the extent of fermentations rather than the type of fermentation. However, further studies are required to appropriately assess this mechanism. Compounds associated to the methylotrophic pathway appeared to explain little variation in CH_4_ emissions, both when comparing within and between diets. Finally, pectin content alone may not be adequate to determine the content of methyl compounds in the rumen, and future studies may benefit in pairing it with other factors to quantify the effects of methyl compounds more fully. Therefore, the results of this study provide the basis for a better understanding of the influence of the diet composition, specifically silage quality, in beef cattle CH_4_ emissions, while shedding light on the importance of the underlying metabolic processes.

## Supplementary Information

Supplementary Information.
