# Supplementary material for: Methane emissions and rumen metabolite concentrations in cattle fed two different silages
Source: Sci Rep. 2022 Mar 31;12:5441. doi: 10.1038/s41598-022-09108-w (PMC8971404; doi:10.1038/s41598-022-09108-w)
Supplement: Supplementary file 1 — Supplementary Information. [file 41598_2022_9108_MOESM1_ESM.pdf]

Supplementary Information for:

# Methane Emissions and Rumen Metabolite Concentrations in Cattle Fed Two Different Silages.

R. Bica[1,2,7*], J. Palarea-Albaladejo[3,6], J. Lima[1,2], D. Uhrin[4], G.A. Miller[1], J.M. Bowen[1], D. Pacheco[5], A. Macrae[2], R.J. Dewhurst[1]

Riccardo Bica
Email: Riccardo.bica@sruc.ac.uk

**This PDF file includes:**

Supplementary Figure 1S
Supplementary Table 1S
Extra References

| | Parameter | Value |
|---|---|---|
| 1 | Data File Name | D:/ 2D_experiments_old_new/ 19/ pdata/ 1/ 2rr |
| 2 | Title | 2D_experiments_old_new. 19.1.2rr |
| 3 | Comment | echo/ antiecho edited HSQC w/ sensitivity improvement w/ adiabatic bilevel decoupling |
| 4 | Origin | Bruker BioSpin GmbH |
| 5 | Owner | chemist |
| 6 | Site | |
| 7 | Instrument | Neo800 |
| 8 | Author | |
| 9 | Solvent | H2O+D2O |
| 10 | Temperature | 298.0 |
| 11 | Pulse Sequence | hsqcedetgpsisp2.3 |
| 12 | Experiment | HSQC-EDITED |
| 13 | Probe | Z168389_0002 (CP2.1 TCI 800S6 H-C/ N-D-05 Z XT) |
| 14 | Number of Scans | 48 |
| 15 | Receiver Gain | 64.0 |
| 16 | Relaxation Delay | 1.5000 |
| 17 | Pulse Width | 12.3200 |
| 18 | Presaturation Frequency | |
| 19 | Acquisition Time | 0.1065 |
| 20 | Acquisition Date | 2020-01-19T15:27:43 |
| 21 | Modification Date | 2020-01-19T15:27:32 |
| 22 | Class | |
| 23 | Spectrometer Frequency | (799.57, 201.07) |
| 24 | Spectral Width | (9615.4, 33181.3) |
| 25 | Lowest Frequency | (-968.2, -956.4) |
| 26 | Nucleus | (1H, 13C) |
| 27 | Acquired Size | (1024, 1024) |
| 28 | Spectral Size | (4096, 2048) |
| 29 | Digital Resolution | (2.35, 16.20) |

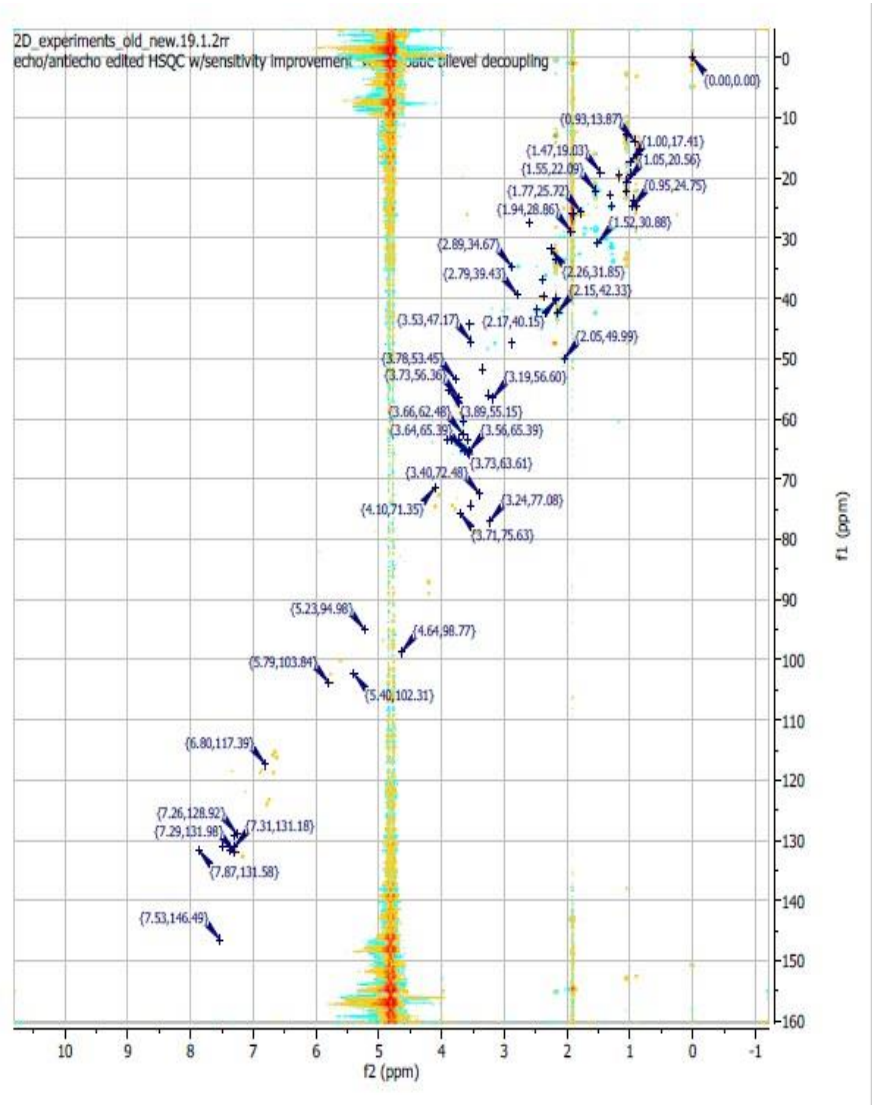


**Supplementary Figure 1S.** 2D HSQC spectra with the $^{1}H$ and $^{13}C$ chemical shifts of the identified metabolites

**Supplementary Table 1S**. Metabolites identified by 2D $^{1}$H-$^{13}$C HSQC and $^{1}$H-$^{1}$H TOSCY spectra and their respective $^{1}$H and $^{13}$C chemical shifts and their RANCM Rank (Joesten and Kennedy, 2019)[(1)]

| Compound | HMDB $^{1}$H and $^{13}$C Chemical Shifts | 2D HSQC/TOCSY $^{1}$H and $^{13}$C Chemical Shifts | COLMARm $^{1}$H and $^{13}$C Chemical Shifts | RANCM Rank |
|---|---|---|---|---|
| 1. Acetate | 1.90/26.08 | 1.91/26.04 | 1.90/25.99 | 3S |
| 2. Butyrate | 2.14/42.34,<br>1.54/22.07,<br>0.91/15.91 | 2.15/42.33,<br>1.55/22.09,<br>0.88/15.96 | 2.14/42.33,<br>1.55/22.08,<br>0.88/15.93 | 3 |
| 3. Propionate | 2.16/33.48,<br>1.03/12.98 | 2.17/33.46,<br>1.04/12.90 | 2.17/33.41,<br>1.04/12.89 | 3 |
| 4. Methanol | 3.37/51.43 | 3.35/51.76 | 3.34/51.57 | 3S |
| 5. Isobutyrate | 1.04/22.00,<br>2.37/39.57 | 1.05/22.09,<br>2.38/39.67 | 1.05/22.10,<br>2.37/39.67 | 3S |
| 6. Ethanol | 1.17/19.59,<br>3.64/60.30 | 1.17/19.59,<br>3.65/60.30 | 1.17/19.49,<br>3.64/60.13 | 3 |
| 7. Isovalerate | 0.89/24.67,<br>1.93/28.84,<br>2.03/49.90 | 0.90/24.67,<br>1.94/28.86,<br>2.05/49.99 | 0.90/24.59,<br>1.94/28.89,<br>2.04/49.94 | 3 |
| 8. Leucine | 0.94/23.57,<br>0.95/24.79,<br>1.70/26.77,<br>1.70/42.59,<br>3.74/56.21 | 0.95/23.62,<br>0.95/24.75,<br>No peak,<br>No peak,<br>3.73/56.36 | 0.94/23.59,<br>0.96/24.75,<br>1.70/26.87,<br>1.70/42.53,<br>3.72/56.11 | 3 |
| 9. Uracil | 5.79/103.71,<br>7.48/146.25,<br>7.56/146.25 | 5.79/103.84,<br>7.53/146.49,<br>No shift | 5.79/103.80,<br>7.53/146.26,<br>No shift | 3 |
| 10. 3-PP | 2.48/41.87,<br>2.87/34.56,<br>7.25/128.79,<br>7.30/131.04,<br>7.35/131.16 | 2.48/41.92,<br>2.89/34.67,<br>7.26/128.92,<br>7.31/131.18,<br>7.36/131.10 | 2.48/41.87,<br>2.88/34.56,<br>7.26/128.79,<br>7.30/131.04,<br>7.36/131.16 | 3 |
| 11. Alanine | 1.49/19.02,<br>3.77/53.55 | 1.47/19.03,<br>3.78/53.45 | 1.47/18.87,<br>3.77/53.22 | 3 |
| 12. Phenylacetate | 3.52/47.17,<br>7.29/131.85,<br>7.29/129.15,<br>7.37/131.51 | 3.53/47.17<br>7.29/131.98<br>7.30/129.24<br>7.36/131.50 | 3.52/47. 17<br>7.29/129.16<br>7.29/131.89<br>7.37/131.37 | 3 |
| 13. Glucose | 3.23/76.95,<br>3.39/72.34,<br>3.52/74.19,<br>3.73/63.35,<br>3.89/63.47,<br>4.63/98.71,<br>5.22/94.93 | 3.24/77.08,<br>3.340/72.48,<br>3.53/74.34,<br>3.73/63.61,<br>3.90/63.53<br>4.64/98.77,<br>5.23/94.97 | N.A,<br>N.A,<br>N.A,<br>N.A,<br>N.A<br>4.64/98.64,<br>5.22/94.83 | 3 |
| 14. Glutamate | 1.77/25.65,<br>2.17/39.90 | 1.77/25.72,<br>2.17/39.91 | 1.78/25.72,<br>2.17/40.05 | 3 |
| 15. Valine | 0.97/19.40,<br>1.03/20.75,<br>2.25/31.89,<br>3.59/ 63.35 | 0.98/19.35,<br>1.04/20.56,<br>2.26/31.85,<br>3.58/63.37 | 0.90/19.37,<br>1.03/20.70,<br>2.27/31.83,<br>3.60/63.08 | 3 |
| 16. Valerate | 0.84/15.82,<br>0.90/15.82,<br>1.28/24.62,<br>1.51/30.77,<br>2.16/40.06 | 0.85/15.40,<br>Peak overlap,<br>1.29/24.75,<br>1.52/30.88,<br>2.17/40.15 | 0.88/15.89,<br>No peak,<br>1.29/24.75,<br>1.51/30.85,<br>2.17/40.12 | 3 |
| 17. Methylamine | 2.59/27.60 | 2.59/27.49 | N.A | 3S |
| 18. Trimethylamine | 2.88/47.47 | 2.88/47.41 | N.A | 3S |
| 19. Succinate | 2.38/36.82 | 2.40/36.85 | N.A | 3S |
| 20. Isoleucine | 0.92/13.91,<br>0.99/17.36,<br>1.45/26.99,<br>1.96/38.68,<br>3.65/62.52 | 0.93/13.87,<br>1.00/17.41,<br>No peak,<br>No peak,<br>3.66/62.48 | N.A | 3 |
| 21. Lactate | 1.31/22.90,<br>4.10/71.37 | 1.32/22.90,<br>4.10/71.35 | N.A | 3 |
| 22. Glycine | 3.54/44.30 | 3.55/44.34 | N.A | 3S |
| 23. Maltose | 3.57/74.38,<br>3.58/77.25,<br>3.62/79.41,<br>3.67/75.67,<br>3.71/75.44, | No peak,<br>No peak,<br>No peak,<br>No peak,<br>3.70/75.63, | N.A | 1 |

| | | | | |
|---|---|---|---|---|
| | 3.80/63.29, 3.83/63.23, 4.64/98.50, 5.22/94.64, 5.39/102.28 | No peak, 3.83/63.45, Peak overlap, Peak overlap, 5.40/102.31 | | |
| 24. Benzoate | 7.47/130.97 7.86/131.44 7.64/133.95 | 7.47/131.10, 7.87/131.58, No peak | N.A | 2 |
| 25. Aspartate | 2.71/39.33, 2.80/39.47, 3.90/55.08 | No peak, 2.79/39.43, 3.89/55.15 | N.A | 2 |
| 26. Dimethylamine | 2.71/37.29 | N.A | N.A | 1S |
| 27. 2-Hydroxyvalerate | N.A | N.A | N.A | N.A |
| 28. Creatinine | 3.03/32.97, 4.05/59.23 | N.A | N.A | 1 |
| 29. Xanthine | 7.92/140.43 | N.A | N.A | 1S |
| 30. Formate | 8.39/172.41 | N.A | N.A | 1S |
| 31. Acetoacetate | 2.27/32.24, 3.43/56.18 | N.A | N.A | 1 |
| 32. 1,3-Dihydroxyacetone | 4.40/67.49 | N.A | N.A | 1S |
| 33. Alloisoleucine | 0.88/16.00, 0.95/13.81, 0.96/16.05, 1.33/28.08, 1.42/28.18, 2.05/38.32, 3.73/61.39 | N.A | N.A | 1 |
| 34. Caffeine | 3.41/27.99, 3.59/29.61, 3.99/33.73, 7.50/141.41 | N.A | N.A | 1 |
| 35. Malonate | 3.11/50.23 | N.A | N.A | 1S |
| 36. Choline | 3.18/56.69, 3.50/70.14, 4.05/58.49 | 3.19/56.60, No peak, No peak, | N.A | 1 |
| 37. Betaine | 3.25/55.85, 3.88/68.64 | 3.25/56.03, No peak | N.A | 1 |
| 38. Creatine | 3.02/39.50, 3.91/56.43 | N.A | N.A | 1 |
| 39. Fumarate | 6.50/138.00 | N.A | N.A | 1S |
| 40. Acetamide | 2.00/22.11 | N.A | N.A | 1S |
| 41. 3-Hydroxyphenylacetate | 3.46/47.00, 6.78/116.16, 6.78/118.81, 6.84/124.10, 7.24/132.64 | No peak, 6.80/117.39, Peak overlap, No peak, No peak | N.A | 1 |
| 42. Fructose | 3.55/66.83, 3.54/65.41, 3.66/65.59, 3.78/70.42, 3.88/72.53, 3.98/72.14, 4.10/77.42 | No peak, 3.56/65.39, 3.64/65.34, No peak, No peak, No peak, No peak | N.A | 1 |

# Extra References

1. Joesten, W. C. & Kennedy, M. A. RANCM: a new ranking scheme for assigning confidence levels to metabolite assignments in NMR-based metabolomics studies. *Metabolomics.* **15**(1) doi: 10.1007/s11306-018-1465-2 (2019).
